# Overexpression of PFTK1 predicts resistance to chemotherapy in patients with oesophageal squamous cell carcinoma

**DOI:** 10.1038/bjc.2012.35

**Published:** 2012-02-14

**Authors:** H Miyagaki, M Yamasaki, H Miyata, T Takahashi, Y Kurokawa, K Nakajima, S Takiguchi, Y Fujiwara, H Ishii, F Tanaka, M Mori, Y Doki

**Affiliations:** 1 Department of Gastroenterological Surgery, Osaka University Graduate School of Medicine, 2-2 Yamadaoka, Suita-shi, Osaka 565-0871, Japan; 2 Department of Surgery, Medical Institute of Bioregulation, Kyushu University, Beppu, Japan

**Keywords:** oesophagus squamous cell carcinoma, PFTK1, immunohistochemistry, chemotherapy, prediction, biological marker

## Abstract

**Background:**

Recently, PFTK1 was identified as a member of the cyclin-dependent kinase family; however, its expression and clinical significance in oesophageal squamous cell carcinoma (ESCC) have not been evaluated.

**Methods:**

PFTK1 expression was initially examined by expression microarray in 77 ESCC patients. Using independent samples of 223 patients, PFTK1 expression was evaluated immunohistochemically to assess the relationship between expression and various clinicopathological parameters. The association between PFTK1 and the response to chemotherapy was also investigated in pretreatment samples of 85 patients who received chemotherapy as first treatment.

**Results:**

Significant upregulation of PFTK1 expression was noted in ESCC compared with normal epithelium. PFTK1 expression was positive in 51.6% (115 out of 223) of the tumours, but did not correlate with any clinicopathological parameter. The 5-year overall survival rate was poorer in patients positive for PFTK1 (43.6%) than those with negative expression (66.2%, *P*<0.001). Uni- and multivariate analyses identified PFTK1 as an independent marker of prognosis (RR=2.428, 95% CI=1.615–3.711, *P*<0.001). Out of 85 biopsy samples, 40 (47.1%) tumours showed PFTK1-positive expression, and the response rate to chemotherapy was significantly lower than PFTK1-negative tumours (27.9% *vs* 72.1%, *P*<0.001).

**Conclusion:**

PFTK1 is not only useful as a prognostic marker, but also as a predictor of the response to chemotherapy.

Oesophageal squamous cell carcinoma (ESCC), the major histological form of oesophageal cancer in East Asian countries, is characterised by poor prognosis and rapid clinical progression. Owing to the high frequency of lymph node metastasis and recurrence, the initial diagnosis is often made when malignancy is in the advanced stage (Shimada *et al*, 2003). Surgery is generally regarded as the standard treatment for these patients; however, the prognosis remains unsatisfactory despite curative resection (Kelsen *et al*, 1998; Medical Research Council Oesophageal Cancer Working Group, 2002). To improve the prognosis, multimodality treatments such as chemotherapy have been vigorously used worldwide. Many clinical trials have been conducted for both neoadjuvant and adjuvant chemotherapy; nevertheless, the results have not been consistent, and no agreement has been reached as to which modality should be employed for advanced oesophageal cancers. Previous studies suggested that neoadjuvant chemotherapy enhance survival in responding patients (Medical Research Council Oesophageal Cancer Working Group, 2002; Gebski *et al*, 2007; Matsuyama *et al*, 2007; Akita *et al*, 2009; Ando *et al*, 2012; Sjoquist *et al*, 2011); yet the reported response rate to chemotherapy is only 19–40% (Kelsen *et al*, 1998; Ancona *et al*, 2001). In addition, most patients have a poor response to chemotherapy, because of the considerable variability and heterogeneity. The non-responders do not only suffer from side effects, but also lose precious time to take advantage of other possible treatments. Thus, accurate prediction of the outcome and response to chemotherapy could allow tailoring the treatment to the individual patient in order to improve outcome and avoid unnecessary treatments.

The PFTK1 gene, also known as PFTAIRE1 or cyclin-dependent kinase (CDK)14, is a new member of the CDK family. The human genome contains 21 genes encoding CDKs (Malumbres *et al*, 2009). The current nomenclature for CDK proteins includes 10 classical CDKs (CDK1-10) and 11 newly proposed family members (CDK11A-20). The roles of 10 ‘classical’ CDKs (CDK1-10) proteins have been investigated to a different extent. CDKs regulate cell cycle progression (CDK1, CDK2, CDK3, CDK4, and CDK6), transcription (CDK7, CDK8, CDK9, and CDK10), differentiation (CDK5), and other processes (Morgan, 2007). Moreover, some CDKs have been implicated in prognosis (Marone *et al*, 1998; Mihara *et al*, 2001; Kim *et al*, 2008) as well as in sensitivity to chemotherapy (Nakayama *et al*, 2009) in human cancers. However, only little information is available on most of the new members of CDKs. PFTK1 promotes the cell cycle (Shu *et al*, 2007) as classical CDKs, and also regulates several pathways and cellular mechanisms as an oncogene (Pang *et al*, 2007; Jiang *et al*, 2009). Recent reports demonstrated that PFTK1 promotes invasiveness and cell motility in hepatocellular carcinoma (HCC) (Pang *et al*, 2007; Leung *et al*, 2011). However, its expression and clinical significance in ESCC has not been yet reported.

In a preliminary study, we found significant upregulation of PFTK1 in ESCC compared with normal epithelial cells in gene expression profile of 21 CDKs. The present study examined the clinical significance of PFTK1 expression and its correlation with the sensitivity to chemotherapy in ESCC patients.

## Patients and methods

### Patients

A total of 77 ESCC patients underwent surgical resection as their first-line treatment between 1992 and 2000 at Kyushu University Hospital at Beppu and the affiliated hospitals, Kurume University Hospital and Kagoshima University Hospital. These patients, whose samples were submitted for expression microarray, were the same patients who participated in our previous study (Kogo *et al*, 2011) and constituted the screening group. A total of 241 patients with primary thoracic ESCC confirmed by histopathological examination, received treatment at Osaka University Hospital between October 1999 and March 2006. All patients were newly diagnosed and had received no prior treatment. All underwent oesophagoscopy and enhanced-computed tomography (CT) from the neck to the abdomen for tumour staging, according to the criteria of the International Union Against Cancer (UICC TNM classification 7th edition (Wittekind, 2010)). In addition, some patients underwent magnetic resonance imaging, endoscopic ultrasonography, positron emission tomography, and bronchial fiberscopy to obtain further information. Of the 241 patients, the first-line treatment was oesophagectomy in 135, chemotherapy with FAP regimen in 98 (Akita *et al*, 2006; Yano *et al*, 2006; Matsuyama *et al*, 2007; Makino *et al*, 2008) and chemoradiotherapy in 8 patients. Out of 98 patients who received chemotherapy, 94 subsequently underwent oesophagectomy whereas the remaining 4 subsequently received radical chemoradiotherapy. All the 8 patients who received chemoradiotherapy underwent oesophagectomy as a second-line treatment. Thus, a total of 237 patients underwent oesophagectomy. Among them, 223 patients underwent curative resection (the resected group) excluding three pathological complete response (CR) cases. Out of the 98 patients who underwent chemotherapy with FAP regimen, we were able to collect biopsy samples containing tumour cells from 85 patients (the biopsied group) (Supplementary Figure S2).

### Treatment protocol and follow up

The basic strategy for treatment of patients with ESCC has been described previously (Yamasaki *et al*, 2010). Subtotal oesophagectomy via right thoracotomy with two- or three-field lymphadenectomy was performed in all patients (Yoshioka *et al*, 2002). Patients with lymph node metastasis at initial diagnosis received neoadjuvant chemotherapy, which consisted of two courses of 5-fluorouracil (5-FU), cisplatin (CDDP), and adriamycin (Akita *et al*, 2006; Yano *et al*, 2006; Matsuyama *et al*, 2007; Makino *et al*, 2008). Furthermore, adjuvant chemotherapy (docetaxel or CDDP plus 5-FU regimen) was provided to patients with larger numbers of pathologically positive lymph nodes (Ando *et al*, 2003). After surgery, the patients were surveyed every 3 months by physical examination and measurement of serum tumour markers (squamous cell carcinoma antigen and carcinoembryonic antigen), every 6 months by enhanced CT scan and abdominal ultrasonography, and annually by endoscopy until tumour recurrence was evident. Patients with tumour recurrence or with non-curative resection received chemo- or chemoradiotherapy as long as their systemic condition permitted. The mean follow-up period after surgery was 45.1 months. The clinicopathological variables were obtained from the medical records and the disease stages was classified in each patient according to the UICC TNM classification seventh edition (Wittekind, 2010).

### Evaluation of effect of treatment

Within 2 weeks after the completion of chemotherapy with FAP regimen, all patients were restaged through endoscopies and enhanced CT scans to evaluate the clinical response to chemotherapy. The clinical response was categorised according to the criteria of the Japanese Society for Esophageal Diseases (Society, 2008) as follows: A CR was defined as total regression of the disease. A CR of the primary tumour represented disappearance of the tumour on CT scan and/or endoscopy. A partial response (PR) was defined as >50% reduction in primary tumour size and lymph node metastasis, as confirmed by CT scan. Progressive disease (PD) was defined as >25% increase in the primary tumour or the appearance of new lesions. Cases that did not meet the criteria of PR or PD were defined as stable disease.

### Immunohistochemical staining

Surgically resected specimens and biopsy samples were fixed in 10% formalin and embedded in paraffin, using conventional techniques. All specimens and samples were cut into 4 *μ*m-thick sections. For resected specimens, one representative slide with the deepest tumour invasion was selected from each patient and subjected to immunohistochemistry as follows. For biopsy samples, serial sections were prepared for haematoxylin and eosin (H&E) and PFTK1 immunohistochemical staining to confirm the inclusion of tumour cells. Immunohistochemistry was conducted as follows: after deparaffinisation in xylene and dehydration in graded ethanol solutions, the tissue sections were heated at 121 °C for 20 min in ethylenediaminetetraacetic acid-tris buffer (pH 9.0) for antigen retrieval. Then endogenous peroxidase activity was blocked by incubation with 30 ml l^−1^ hydrogen peroxide for 20 min. After overnight incubation with rabbit polyclonal primary antibody PFTK1 (HPA015267, Sigma-Aldrich Co., St. Louis, MO, USA, dilution 1 : 500) at 4 °C, staining was performed by the labelled streptavidin–biotin method. For the negative control, the primary antibody was omitted from the immunohistochemical reaction. HCC was used as a positive control. Staining for PFTK1 in each ESCC sample was judged positive when >10% of the cancer cells in the section were immunoreactive to PFTK1, or otherwise negative when only ⩽10% of the cells were positive. All slides were assessed independently by two pathologists and then by conference in case of disagreement. Both pathologists were blinded to the clinicopathological data.

### Reverse transcriptase–polymerase chain reaction (RT–PCR)

Total RNA from frozen tumour tissue samples were extracted using Trizol reagent (Invitrogen, Carlsbad, CA, USA) following the instructions supplied by the manufacturer. Total RNA was reverse transcribed to cDNA in a 20-*μ*l volume using Reverse Transcription System (A3500 Promega, Madison, WI, USA). The reaction condition set based on the recommendation by the manufacturer. RT–PCR was carried out in a reaction mixture containing 2 *μ*l of cDNA, 12.5 *μ*l AmpliTaq GOLD (Applied Biosystems, Foster City, CA, USA) and 10.5 *μ*l water. The cycling conditions were 95 °C for 5 min followed by 35 cycles of 95 °C for 30 s, 60 °C (58 °C for GAPDH) for 30 s, 72 °C for 2 min, and a final extension at 72 °C for 7 min. Equal amounts of PCR products were electrophoresed on 1.5% agarose gels and visualised by ethidium bromide staining. Primers were designed as described previously (Dohadwala *et al*, 2010; Tsuji *et al*, 2010). Primer sequences were as follows: human PFTK1 5′-CCAAGGAGTTGCTGCTTTTC-3′ (sense) and 5′-GAATGAACTCCAGGCCATGT-3′ (anti-sense); human GAPDH 5′-CAACTACATGGTTTACATGTT-3′ (sense) and 5′-GCCAGTGGACTCCACGAC-3′ (anti-sense).

### Laser microdissection (LMD)

Tissues were collected from 77 ESCC cases (screening group) for LMD. For this purpose, ESCC tissues were microdissected using the LMD system (Leica Laser Microdissection System, Leica Microsystems, Wetzlar, Germany), as described previously (Nishida *et al*, 2005). For LMD, 5 *μ*m frozen sections were fixed in 70% ethanol for 30 s, stained with H&E, and dehydrated as follows: 5 s each in 70%, 95%, and 100% ethanol and a final 5 min in xylene. Sections were air-dried, then microdissected using the LMD system. The target cells were excised, with each section containing at least 100 cells, bound to transfer film, and total DNA and RNA extracted.

### Expression microarray

Expression microarray was conducted using samples from 77 ESCC cases (screening group). The resected cancer tissues were immediately cut and embedded in Tissue-Tek OCT medium (Sakura, Tokyo, Japan), frozen in liquid nitrogen, and kept at −80 °C until RNA extraction. These samples were obtained using LMD. Following isolation of RNA, cRNA was synthesised from 8.0 *μ*g total RNA as described previously (Inoue *et al*, 1995). We used the commercially available Human Whole Genome Oligo DNA Microarray Kit (Agilent Technologies, Santa Clara, CA, USA). Cyanine-labelled cRNA was prepared using T7 linear amplification, as described in the Agilent Low RNA Input Fluorescent Linear Amplification kit manual (Agilent Technologies). Labelled cRNA was then fragmented and hybridised to an oligonucleotide microarray (Whole Human Genome 4 × 44 Agilent G4112F). Fluorescence intensity was determined with Agilent DNA microarray scanner and analysed using G2567AA Feature Extraction Software version A7.5.1 (Agilent Technologies), which employs the LOWESS (locally weighted linear regression curve fit) normalisation method (Quackenbush, 2002). This microarray study followed MIAME guidelines issued by the Microarray Gene Expression Data group (Brazma *et al*, 2001). Further analyses were performed using GeneSpring version 7.3 (Silicon Genetics, San Carlos, CA, USA).

### Statistical analysis

Correlations between PFTK1 expression and various clinicopathological parameters were each evaluated by the *χ*^2^-test and Fisher's exact probability test. Differences in mRNA expression levels were compared using Student's *t*-test. Prognostic variables were assessed by log-rank test. Overall survival (OS), recurrence-free survival (RFS), and progression-free survival (PFS) rates were analysed by the Kaplan and Meier method. Cox's proportional hazard regression model was used to analyse the independent prognostic factors. These analyses were carried out using JMP version 8.0.1 (SAS Institute, Cary, NC, USA) for Windows. A *P*-value of <0.05 denoted the presence of statistical significance.

## Results

### Expression of CDKs in gene expression profile

**Figure 1:**
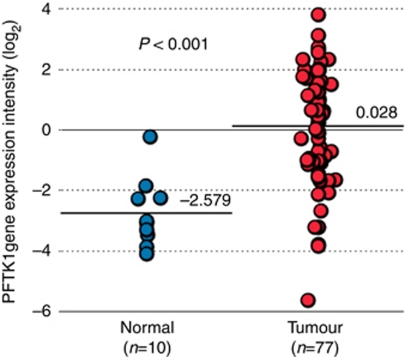
Intensity of PFTK1 expression in normal tissues and ESCC from DNA array data. Symbols represent samples of different patients. Horizontal lines represent the mean expression level for the group.

Gene expression profile identified 21 members of the CDK family. Table 1 lists the fold changes in tumour cell intensity in binary logarithm relative to normal tissue intensity. Upregulation of PFTK1 expression was noticeable in tumour tissue compared with the normal tissue (fold change 2.607, *P*<0.001, Figure 1). Significant upregulation was also noted in CDK1, CDK4, and CDK18 (CDK1: fold change 1.316, CDK4: 1.391, CDK18: 1.450; *P*<0.001, each) whereas significant downregulation was evident in CDK7 (fold change −1.436, *P*<0.001).

### Immunohistochemical analysis of PFTK1 expression in ESCC

**Figure 2:**
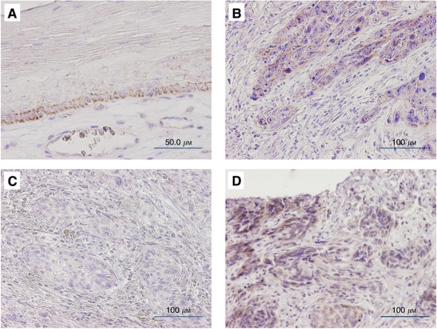
Representative examples of immunohistochemical staining for PFTK1. (**A**) Normal squamous epithelium. Note the lack of staining except in a few basal cells. (magnification × 200). (**B**) PFTK1-positive ESCC. Note that the staining is mainly in the cytoplasm of tumour cells (magnification × 200). (**C**) PFTK1-negative ESCC. Note almost no appreciable staining of tumour cells (magnification × 200). (**D**) Biopsy sample of ESCC positive for PFTK1 staining (magnification × 200).

A total of 223 cases (resected group) that contained both cancerous and non-cancerous lesions were evaluated for PFTK1 protein expression by immunohistochemical analysis, whereas 3 cases were excluded because of pathological CR to neoadjuvant chemotherapy. None of the normal squamous epithelium showed significant levels of PFTK1 expression, although some basal cells showed faint immunostaining in the cytoplasm (Figure 2A). Of the 223 tumours, 115 (51.6%) were PFTK1-positive mainly in the cytoplasm of tumour cells (Figure 2B), whereas the remaining 108 (48.4%) were negative (Figure 2C). The positive staining was almost homogeneous at single cancer nests and among different areas (surface, central, and deepest areas) of the cancer lesion. The grading of immunostained sections was almost identical by the two pathologists, with interobserver variation of <5%.

### PFTK1 mRNA and protein expression in ESCC clinical tissue specimens

Next, we assessed the consistency between the mRNA expression and protein expression of PFTK1 in resected tumours and adjacent non-cancerous tissues from randomly selected 6 of the 223 patients (resected group). PFTK1 mRNA expression was not observed in all six non-cancerous tissue samples; however, three of the six tumours had strong expressions of PFTK1 mRNA, consistent with each sample's immunohistochemical analysis (Supplementary Figure S1).

### PFTK1 expression and clinicopathological characteristics of the resected group

**Figure 3:**
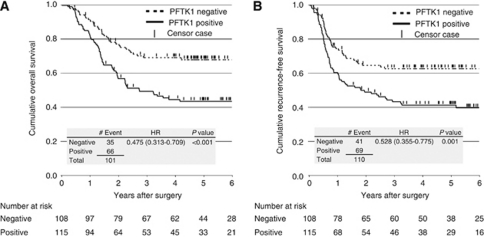
(**A**) Overall and (**B**) RFS curves according to PFTK1 expression. Survival curves were analysed by the Kaplan–Meier method and differences between two groups were evaluated by log-rank test.

Table 2 lists the correlations between PFTK1 expression and various clinicopathological parameters of the 223 patients who underwent curative resection (resected group). They included 24 females and 199 males, aged between 38 and 84 years (median, 64 years). The median follow-up period of the 223 patients after surgery was 44.7 months (range, 1.6–120.0 months), and 101 patients (45.3%) died (81 of the disease and 20 of other causes) during the follow-up period. None of the patients died of post-operative complications. Age, gender, tumour location, histology, pT, pN, pM, and pStage, did not correlate with PFTK1 expression. A total of 88 patients received neoadjuvant therapy, and their response rate (CR and PR) was 48.9%. The rate of PFTK1(−) cases (*n*=38) was 63.2%, which was significantly higher than PFTK1(+) cases (*n*=50; 38.0%, *P*=0.031). The 5-year OS rate of the 223 patients was 54.9%, and the 5-year RFS rate was 51.7%. Patients with PFTK1(+) tumours (*n*=115) showed significantly poorer OS and RFS than those with PFTK1(−) tumours (*n*=108) (5-year OS: 43.6% *vs* 66.2%, *P*<0.001, 5-year RFS; 41.4% *vs* 62.8%, *P*=0.001, Figure 3). Disease recurrence was diagnosed after surgery in 90 (40.4%) patients, and their median survival time and time to recurrence was 16.9 and 14.8 months, respectively. Recurrence was more frequent in patients with PFTK1(+) tumours (PFTK1(+): 57/115(49.6%), PFTK1(−): 33/108 (30.6%), *P*=0.004). However, the site of recurrence was not different between PFTK1(+) and (−) patients (data not shown).

Univariate analysis showed that OS correlated significantly with pT, pN, pM, and PFTK1 expression (Table 3). Multivariate analysis using the above four statistically significant parameters (*P*<0.05) identified PFTK1 as an independent prognostic factor, in addition to pT, pN, and pM (Table 3).

### PFTK1 expression and clinicopathological characteristics in biopsied group

Finally, we analysed the results of immunohistochemical staining of 85 biopsy samples from patients who received chemotherapy with FAP regimen as first-line treatment (biopsied group). The median follow-up period of the group was 41.9 months (range, 2.3–117.1 months), and 48 (56.8%) patients died during the follow-up period. Disease progression was diagnosed in 40 (47.1%) patients. The median survival time and time to progression were 28.7 and 13.8 months, respectively. Of these, 40 (47.1%) were PFTK1-positive (Figure 2D), whereas the remaining 45 (52.9%) were negative. Table 4 lists the clinical parameters of these patients. Age, gender, location of tumour, histological grade, cT, cN, cM, and cStage did not correlate with PFTK1 expression. The response rate to chemotherapy was 50.6% for all cases. The response rate in PFTK1(+) was 27.9%, which was significantly lower than in PFTK1(−) patients (72.1%, *P*<0.001). A pathologically CR was observed in three PFTK1(−) cases. As shown in Supplementary Figure S3, PFTK1(+) patients had a significantly poorer prognosis with regard to OS and PFS (5-year OS: 32.5% *vs* 54.4%, *P*=0.042, 5-year PFS: 18.8% *vs* 52.7%, *P*=0.012).

Comparison of biopsies and resected specimens showed 68.3% of PFTK1(−) cases of the biopsied group were also judged as PFTK1(−) by examination of the resected specimens (Table 4). There were no differences in the response to chemotherapy and in RFS between the converted cases and non-converted cases (data not shown). On the other hand, 86.5% of PFTK1(+) patients of the biopsied group were judged as PFTK1(+). Similarly, there was no difference in the response to chemotherapy between the converted cases and non-converted cases (data not shown).

## Discussion

Some of the CDKs have been implicated in human cancers. CDK1, CDK4, and CDK6 have a diagnostic value in various cancers (Simon *et al*, 2002; Semczuk and Jakowicki, 2004; Hansel *et al*, 2005; Kim *et al*, 2008; Nakayama *et al*, 2009; Poomsawat *et al*, 2010). CDK2 expression or activity has been used as a marker for the prognosis of breast (Kim *et al*, 2008), ovarian (Marone *et al*, 1998), and oral (Mihara *et al*, 2001) cancers. Our study of PFTK1 expression in resected specimens from 223 ESCC patients who underwent curative resection showed PFTK1 expression was a significant marker of poor prognosis and an independent prognostic factor, in addition to pT, pN, and pM.

Moreover, the expression of PFTK1 correlated with the response to chemotherapy. Multimodality therapy in ESCC patients is generally accepted, but many studies have reported only modest improvement of survival, and that survival benefits were noted only in responders to chemotherapy (Kelsen *et al*, 1998; Ancona *et al*, 2001; Tepper *et al*, 2008; Akita *et al*, 2009). Our results showed PFTK1 protein expression in not only resected cancer tissues but also in ESCC biopsy samples obtained before the initiation of treatment, and that this parameter was a predictor of the response to chemotherapy. We analysed the relationship between the clinical outcomes, such as prognosis and response to chemotherapy, and protein expression determined by immunohistochemistry rather than mRNA expression by PCR, based on consideration of the following factors. First, immunohistochemistry was superior to PCR with regard to the handling of tissue sample based on the stability of the protein compared with the mRNA. Second, there was no need to purify biopsy samples by microdissection technique for accurate measurement of gene expression. Microdissection requires not only extra time and effort but also a larger biopsy sample.

Our results identified some cases in which the status of PFTK1 expression was different before and after chemotherapy. About 90% of patients who were judged as PFTK1(+) on the basis of examination of pretreatment biopsy samples were also PFTK1(+) in the post-chemotherapy resected samples. However, over 30% of patients judged as PFTK1(−) in the pretreatment samples became PFTK1(+) in the post-chemotherapeutic resected sample. This finding suggests that administration of chemotherapy seems to alter PFTK1 expression. However, it is possible that a small portion of the cells was actually positive, and that these cells were unmasked by chemotherapy. Indeed, patients, who were judged negative in pretreatment biopsy and positive in resected specimen, tended to have poorer prognosis, albeit statistically insignificant, than those who did not show such change in judgment (5-year OS: 35% *vs* 60%). The results indicate possible involvement of PFTK1 activation in the development of chemoresistance.

Following the identification of PFTK1 as a member of the CDK family, several studies characterised its physiological function and biological importance. Recent studies reported that PFTK1 enhanced incoming Wnt signals (Davidson *et al*, 2009; Niehrs and Shen, 2010), which might relate with the malignancy. With regard to cancer cells, Pang *et al* (2007) reported the role of PFTK1 in cellular invasiveness and motility of HCC cells. To our knowledge, however, the relevance of PFTK1 to the responses to chemotherapy has not been reported. Our study is the first report on this issue but it did not examine how PFTK1 alters chemosensitivity. Further analysis of the mechanism(s) of PFTK1-induced changes in chemosensitivity is recommended.

Our results indicated that PFTK1 was not only a predictor of the responses to chemotherapy but also a potential target of molecular-targeted therapy. Recently, several types of CDK inhibitors were introduced as a novel class of chemotherapeutic agents and expected to improve the effect of cancer treatment (Diaz-Padilla *et al*, 2009; Krystof and Uldrijan, 2010; Le Tourneau *et al*, 2010; Schwartz *et al*, 2011). Some of these agents target a specific type of CDK, but most target multiple CDKs including PFTK1 (Caligiuri *et al*, 2005). Recent studies reported that some of the CDK inhibitors do not only have anticancer properties but also lessened resistance to chemotherapy (Luo *et al*, 2010). Our results also suggested that a CDK inhibitor would be useful in the treatment of ESCC by enhancing the sensitivity to chemotherapy especially in patients with PFTK1(+) tumours, in support of previous reports (Schwartz *et al*, 2002).

The limitations of this study include the limitations of immunohistochemical staining, such as its semiquantitative nature and interobserver variation, all of which may have affected the association between PFTK1 expression and survival. The second limitation is that PFTK1 expression detected in endoscopic biopsy samples may not be representative of the entire tumour, because of tumour heterogeneity. As there is only 70% agreement between PFTK1 expression and the response to chemotherapy, additional larger prospective studies are needed to standardise and optimise methodologies for PFTK1 analysis, and to establish a predictive biological profile. Despite these limitations, however, our results are the first to indicate the usefulness of PFTK1 expression as an independent predictor of the response to chemotherapy in ESCC.

In conclusion, the present study demonstrated that PFTK1 is a novel marker of prognosis of patients with ESCC, which is independent of the traditional TNM classification. PFTK1 expression in biopsy samples also may be a predictor of chemosensitivity in ESCC patients. The use of such marker could allow clinicians to stratify the treatment of ESCC to individual patients. Further studies are needed to evaluate the mechanisms of increased PFTK1 expression and to determine whether targeting this member of CDK family, like other members, is a suitable strategy against ESCC.

## Figures and Tables

**Table 1:** Mean expression level of cyclin-dependent kinases in expression array (*n*=77)

**Gene symbol**	**Fold change**	**(95% CI)**	***P*-value**
CDK1	1.316	(1.132 to 1.504)	<0.001
CDK2	0.511	(0.346 to 0.677)	0.106
CDK3	0.337	(0.207 to 0.466)	0.169
CDK4	1.391	(1.235 to 1.550)	<0.001
CDK5	0.279	(0.147 to 0.479)	0.388
CDK6	0.751	(0.431 to 1.073)	0.203
CDK7	−1.436	(−1.576 to −1.230)	<0.001
CDK8	−0.650	(−0.790 to −0.510)	0.008
CDK9	0.235	(0.137 to 0.374)	0.297
CDK10	−0.282	(−0.502 to −0.066)	0.510
CDK11A	0.741	(0.573 to 0.911)	0.017
CDK11B	0.982	(0.795 to 1.177)	0.004
CDK12	0.789	(0.622 to 0.958)	0.007
CDK13	−0.328	(−0.490 to −0.170)	0.320
PFTK1 (CDK14)	2.607	(2.231 to 2.983)	<0.001
CDK15	−0.249	(−0.411 to −0.087)	0.429
CDK16	−0.084	(−0.245 to 0.054)	0.795
CDK17	0.354	(0.166 to 0.542)	0.328
CDK18	1.450	(1.225 to 1.680)	<0.001
CDK19	−0.493	(−0.656 to −0.330)	0.101
CDK20	0.036	(−0.275 to 0.342)	0.960

Abbreviation: CI=confidence interval.

Fold change indicates the binary logarithm of the tumour/normal ratio of the expression level.

**Table 2:** Correlation between various clinicopathological parameters and PFTK1 expression in resected specimen

	**PFTK1 Expression**		
**Parameters**	**Positive (*n*=115)**	**Negative (*n*=108)**	***P*-value**
*Age*			
<64	52	58	0.229
⩾64	63	50	
			
*Gender*			
Male	102	97	0.832
Female	13	11	
			
*Location of tumour*			
Upper, middle^a^	76	65	0.405
Lower^a^	39	43	
			
*Histological grade*			
G1	26	29	0.535
G2, G3	89	79	
			
*pT*			
T1-2	53	46	0.686
T3-4a	62	62	
			
*pN*			
N0-1	76	73	0.887
N2-3	39	35	
			
*pM*			
M0	103	98	0.825
M1(LYM)	12	10	
			
*pStage*			
I, II	57	49	0.592
III, IV	58	59	
			
*Neoadjuvant therapy*			
Yes	50	38	0.210
No	65	70	
			
*Effect of neoadjuvant chemotherapy*			
CR, PR	19	24	0.031
SD, PD	31	14	

Abbreviations: CR=complete response; PD=progressive disease; PR=partial response; SD=stable disease; UICC TNM=the International Union Against Cancer.

a Upper, Middle and lower thoracic oesophagus. Histological grade, pT, pN, pM, and pStage were according to UICC TNM classification 7th edition (Wittekind, 2010).

**Table 3:** Univariate and multivariate survival analyses of overall survival by Cox’s proportional hazard model

		**Univariate analysis**			**Multivariate analysis**		
**Parameter**	***n***	**RR**	**95% CI**	***P*-value**	**RR**	**95% CI**	***P*-value**
*PFTK1*							
Positive/negative	115/108	2.106	(1.411–3.196)	<0.001	2.428	(1.615–3.711)	<0.001
							
*Age*							
⩾64/<64	113/110	1.194	(0.809–1.771)	0.372			
							
*Gender*							
Female/male	28/214	0.619	(0.277–1.195)	0.164			
							
*Location of tumour*							
Upper, Middle/lower^a^	141/82	0.992	(0.666–1.501)	0.971			
							
*Histological grade*							
G2, G3/G1	168/55	1.374	(0.865–2.281)	0.184			
							
*pT*							
T3-4/T1-2	124/99	2.320	(1.534–3.590)	<0.001	1.738	(1.122–2.747)	0.013
							
*pN*							
N2, 3/N0, 1	74/149	4.027	(2.711–6.006)	<0.001	3.348	(2.167–5.181)	<0.001
							
*pM*							
M1(LYM)/M0	22/201	3.199	(1.850–5.239)	<0.001	2.044	(1.163–3.421)	0.014

Abbreviations: CI=confidence interval; UICC TNM=the International Union Against Cancer.

a Middle, lower, and upper thoracic oesophagus. Histological grade, pT, pN, pM, and pStage (pathological classification) were according to UICC TNM classification 7th edition (Wittekind, 2010).

**Table 4:** Correlation between various clinical parameters, clinical outcome, and PFTK1 in biopsy samples

	**PFTK1 Expression**		
	**Positive (*n*=40)**	**Negative (*n*=45)**	***P*-value**
*Age*			
<64	15	23	0.275
⩾64	25	22	
			
*Gender*			
Female	7	8	1.000
Male	33	37	
			
*Location of tumour*			
Upper, Middle^a^	26	25	0.506
Lower^a^	14	20	
			
*Histological grade*			
G1	8	8	1.000
G2, G3	32	37	
			
*cT*			
T1-2	11	10	0.621
T3-4a	29	35	
			
*cN*			
N0-1	28	29	0.648
N2-3	12	16	
			
*cM*			
M0	27	37	0.137
M1	13	8	
			
*cStage*			
I, II	5	8	0.559
III, IV	35	37	
			
*Effect of chemotherapy*			
CR, PR	12	31	<0.001
SD, PD	28	14	
			
*Curability of resection*			
Curative	31	39	0.415
Non-curative	6	5	
No resection	3	1	
			
*PFTK1 expression of resected specimen*			
Positive	32	13	<0.001
Negative	5	28	
pCR	0	3	

Abbreviations: pCR=pathological complete response; UICC TNM=the International Union Against Cancer.

a Middle, lower, and upper thoracic oesophagus. Histological grade, cT, cN, cM, and cStage were according to UICC TNM classification 7th edition (Wittekind, 2010).
