# Supplementary material for: Overexpression of PFTK1 predicts resistance to chemotherapy in patients with oesophageal squamous cell carcinoma
Source: Br J Cancer. 2012 Feb 14;106(5):947–54. doi: 10.1038/bjc.2012.35 (PMC3305960; doi:10.1038/bjc.2012.35)
Supplement: Supplementary Figures [file bjc201235x1.ppt]

## Slide 1
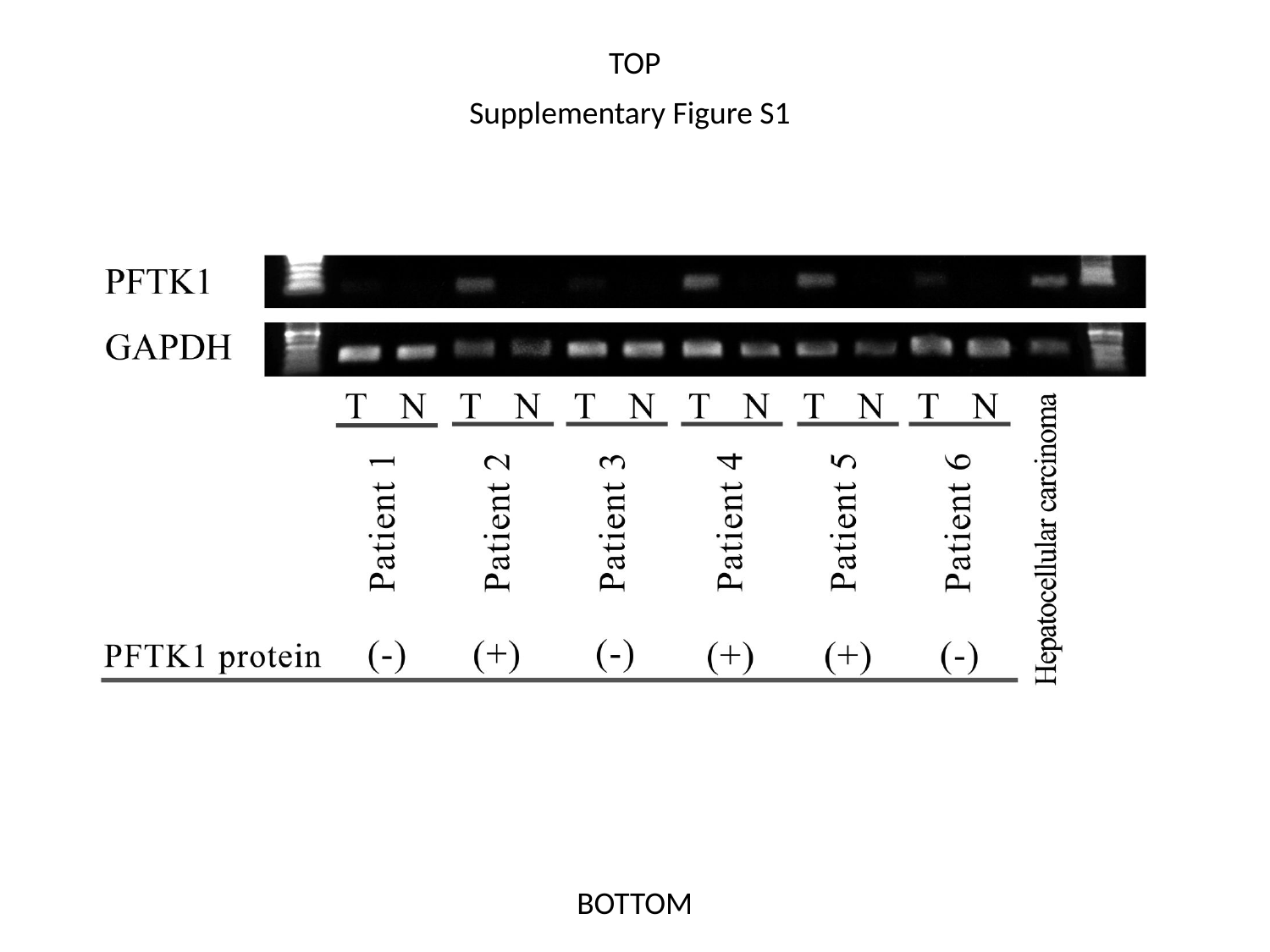

TOP
Supplementary Figure S1
BOTTOM

## Slide 2
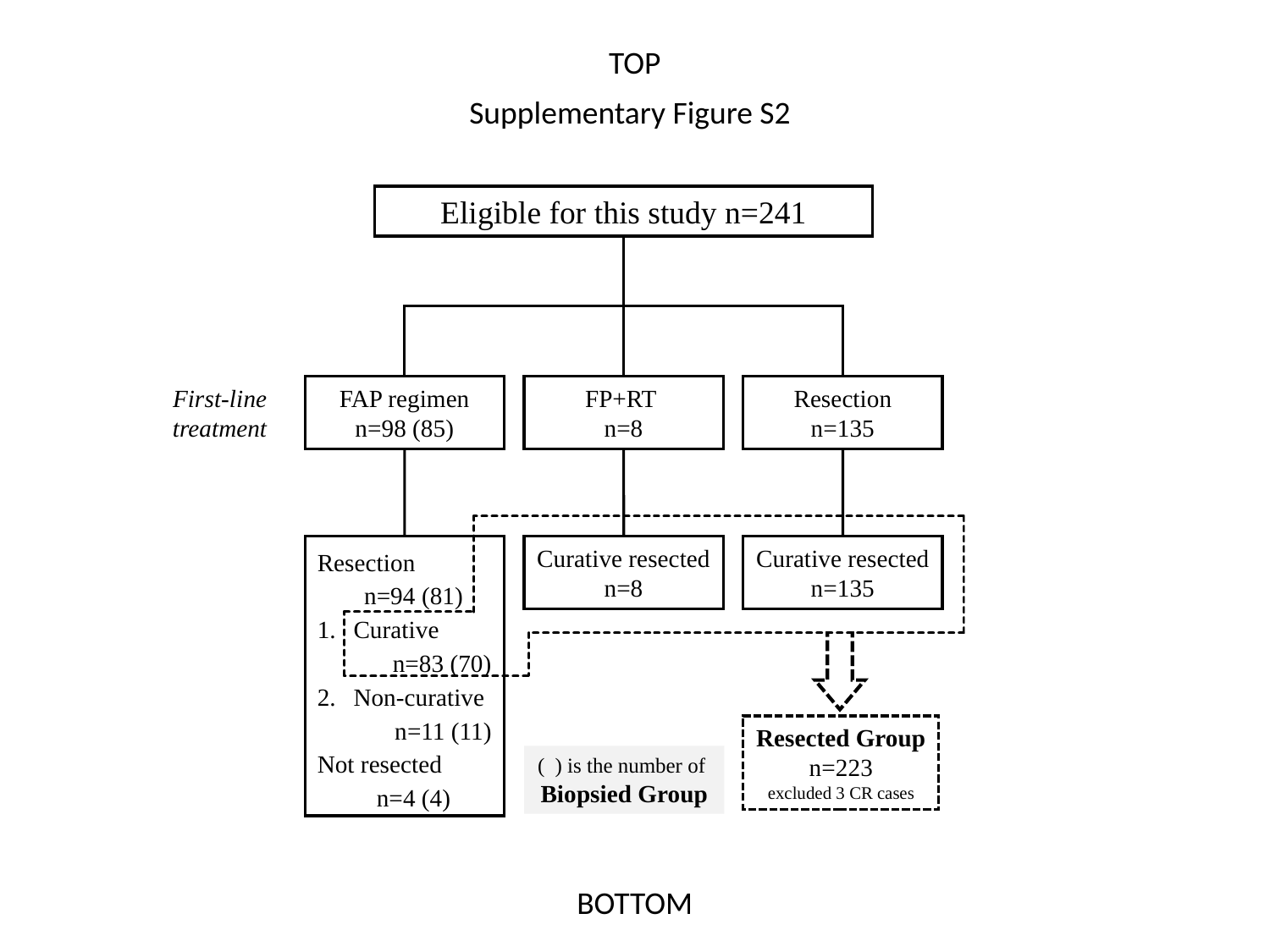

TOP
Supplementary Figure S2
Eligible for this study n=241
FAP regimen n=98 (85)
FP+RT
n=8
Resection
n=135
Curative resected
n=4
Resection
 n=94 (81)
 Curative
 n=83 (70)
 Non-curative
 n=11 (11)
Not resected
 n=4 (4)
Curative resected
n=8
Curative resected
n=135
Resected Group
n=223
excluded 3 CR cases
( ) is the number of
Biopsied Group
First-line
treatment
BOTTOM

## Slide 3
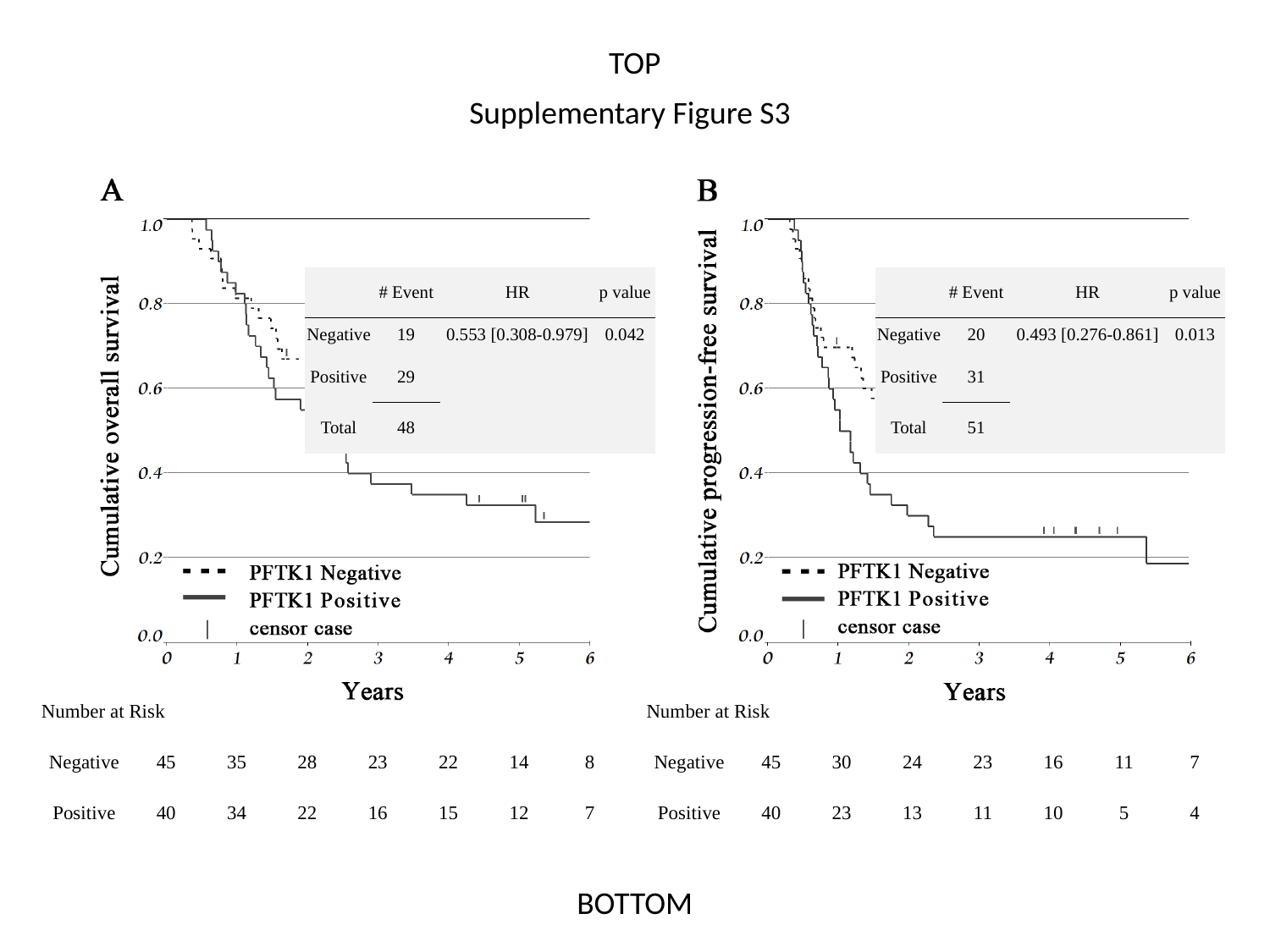

TOP
Supplementary Figure S3
| | | | |
| --- | --- | --- | --- |
| | # Event | HR | p value |
| Negative | 19 | 0.553 [0.308-0.979] | 0.042 |
| Positive | 29 | | |
| Total | 48 | | |
| | | | |
| | | | |
| --- | --- | --- | --- |
| | # Event | HR | p value |
| Negative | 20 | 0.493 [0.276-0.861] | 0.013 |
| Positive | 31 | | |
| Total | 51 | | |
| | | | |
| Number at Risk | | | | | | | | |
| --- | --- | --- | --- | --- | --- | --- | --- | --- |
| | Negative | 45 | 35 | 28 | 23 | 22 | 14 | 8 |
| | Positive | 40 | 34 | 22 | 16 | 15 | 12 | 7 |
| Number at Risk | | | | | | | | |
| --- | --- | --- | --- | --- | --- | --- | --- | --- |
| | Negative | 45 | 30 | 24 | 23 | 16 | 11 | 7 |
| | Positive | 40 | 23 | 13 | 11 | 10 | 5 | 4 |
BOTTOM
