# Supplementary material for: Overexpression of PFTK1 predicts resistance to chemotherapy in patients with oesophageal squamous cell carcinoma
Source: Br J Cancer. 2012 Feb 14;106(5):947–54. doi: 10.1038/bjc.2012.35 (PMC3305960; doi:10.1038/bjc.2012.35)
Supplement: Supplementary Figure Legends [file bjc201235x2.doc]

Supplementary Figure S1: Assessment of PFTK1 mRNA and protein expression levels in six samples randomly selected from 223 ESCC patients.

Supplementary Figure S2: A flow diagram detailing evaluation of the resected group (n=223) and the biopsied group (n=85).

Supplementary Figure S3: (A) Overall and (B) progression-free survival curves according to PFTK1 expression in 85 patients evaluated by biopsy samples analyzed by the Kaplan–Meier method. Differences between the two groups were evaluated by the log-rank test.
